# Studies on the Processing of Fine Dusts from the Electric Smelting of Ilmenite Concentrates to Obtain Titanium Dioxide

**DOI:** 10.3390/ma15238314

**Published:** 2022-11-23

**Authors:** Almagul Ultarakova, Zaure Karshyga, Nina Lokhova, Azamat Yessengaziyev, Kaisar Kassymzhanov, Arailym Mukangaliyeva

**Affiliations:** The Institute of Metallurgy and Ore Beneficiation, Satbayev University, Almaty 050013, Kazakhstan

**Keywords:** ilmenite concentrate, dusts, waste, purification, sublimate, pyrohydrolysis, silicon, titanium dioxide

## Abstract

This article presents studies on the ammonium fluoride processing of dusts from the reduction smelting of ilmenite concentrate with separation of silicon to obtain titanium dioxide. Optimal conditions for pyrohydrolysis of titanium fluorides were determined. The effects of temperature and duration on the process were studied. The optimal conditions for pyrohydrolysis of titanium fluorides were a temperature of 600 °C and duration of 240–300 min. The degree of titanium fluoride conversion to titanium oxide was 99.5% at these conditions. Titanium dioxide obtained by pyrohydrolysis of titanium fluorides was purified from iron, chromium, and manganese impurities. The effect of hydrochloric acid solution concentration, S:L ratio, and the process duration on the purification degree of titanium fluoride pyrohydrolysis was studied. The following optimum purification conditions were determined: hydrochloric acid solution concentration 12.5–15 wt%, temperature 25–30 °C, S:L = 1:6÷8, duration 20–30 min. The purified titanium dioxide consisted mainly of anatase. The pigmented titanium dioxide of rutile modification with 99.8 wt% TiO_2_ was obtained after calcination at 900 °C for 120 min.

## 1. Introduction

Titanium and its compounds are important components of modern production. The properties of titanium, including high strength, good corrosion resistance, and s light weight, make it indispensable in such areas of application as aerospace, shipbuilding, chemical manufacture, military, medical care, etc. China, Japan, Russia, Kazakhstan, USA, and Ukraine are the largest producers of titanium sponge [1,2,3,4]. Ilmenite concentrate is used as raw material for titanium production during reduction electric smelting resulting in production of titanium slag and substandard pig iron. The charge for smelting is fed in a loose state, accompanied by a large dust entrainment. The dust cannot be returned to the smelting process or fed into the chlorinators due to its high silica content so it is stored together with other solid waste in specially designated landfill areas.

The silica content in the ore-thermal smelting dusts of ilmenite concentrates can be in the range of 5–20 wt%. They also contain iron oxides up to 30 wt%. Considerable amounts of titanium dioxide are lost together with the dust generated in the electric smelting process of ilmenite concentrate. Its content therein reaches 50 wt%, and its additional extraction from the dust waste will enable not only loss reduction, but also the ability to obtain additional commercial products.

There is no effective integrated processing of titanium production waste in the world. Available technologies for the production of titanium products are aimed at processing traditional mineral raw materials, the majority of which are ilmenite ores and concentrates.

Of all the mined titanium ore, 5% goes to the production of metallic titanium, and about 90–95% of the mined titanium raw materials are processed into pigment titanium dioxide TiO_2_, which is in special demand and has a high degree of purity. The superiority of TiO_2_ as a white pigment is mainly due to its high refractive index and consequently its light-scattering ability which provide excellent concealment and brightness. Titanium dioxide is non-toxic and harmless; it is used in solar cells [5], fuel cells, chemical current sources, protective and optical coatings, as a photocatalyst for water purification [6], in medicine as a material with antibacterial and antiviral effects [7], for air purification and disinfection in public places [8], for creation of self-cleaning surfaces [9], in photoelectrochemical water decomposition reactions to produce hydrogen [10,11], and in photocatalysts for destruction of toxic organic compounds [12,13,14]. Titanium dioxide nanomaterials doped with nitrogen and fluorine have a higher photocatalytic activity [15,16].

The existing industrial technologies for producing titanium dioxide are sulfuric acid and chlorine methods, which were introduced in the middle of the twentieth century in a number of countries. The titanium-containing raw material is treated with concentrated sulfuric acid in the sulfuric acid process, and a sulfate solution is obtained. Titanium dioxide is precipitated during its hydrolytic decomposition [17,18,19,20,21]. Under the chlorine technology, first, rutile is subjected to the action of chlorine gas, with the formation of titanium tetrachloride, which is then converted into a pigment at high temperature in a mixture of air and oxygen [22]. The main disadvantage of sulfuric acid technology is the formation of a large amount of waste solutions, the disposal of which is difficult. It is necessary to use only high-quality rutile in the chlorine technology, and its production requires additional preliminary preparation of titanium raw materials.

Recently, ammonium fluorine processing has become one of the promising methods for processing titanium-containing raw materials. The fundamentals of the ammonium fluoride method for processing titanium-containing raw materials were laid by Svendsen and described in his patents [23,24]. The methods consist of heating and fluorination of rutile or ilmenite in a mixture with ammonium fluoride or bifluoride, followed by sublimation of titanium fluoride compounds, their decomposition, and the production of titanium dioxide.

The reagent for fluorination, ammonium bifluoride, under normal conditions does not pose a significant environmental hazard, has high chemical activity, and a number of technologically favorable physical and chemical properties, such as a melting point of 126.2 °C and a boiling point of 238 °C, which are accompanied by decomposition into NH_3_ and HF, and good solubility in water at 434 g/dm^3^ [25].

The physicochemical basis of the fluorination process with ammonium bifluoride is that oxygen-containing compounds of transitional and many non-transitional elements, when interacting with NH_4_HF_2_, form ammonium fluoro- or oxofluorometallates, which are very convenient for processing [26], the physicochemical properties of which ensure the solubility of products and the possibility of separating mixtures by sublimation. The great advantage of these complex salts is that the selective tendency to sublimation or to thermal dissociation to non-volatile fluorides, which guarantees a deep separation of the components, and the stepwise elimination of NH_4_F vapors makes it possible to collect the desublimate of the latter and use it in a closed cycle.

As noted above, silicon may concentrate in the dusts of electric smelting of ilmenite concentrates during their formation, and its presence does not allow them to return to the smelting process. Previous studies on ammonium fluoride treatment of cakes obtained from nitric acid leaching of titanium production sludge with ammonium bifluoride have shown the potential for separating silicon and beneficiating the residue with titanium [27,28].

In [29], the fluorination of titanium slag with ammonium hydrofluoride resulted in titanium dioxide with admixtures of other oxides left in the solid product after sublimation of silicon hexafluoride. Further separation of titanium from other components is carried out using leaching with a solution of ammonium hydrofluoride. Titanium is precipitated from the solution with ammonia water. The content of TiO_2_ in the resulting product is more than 90 wt%. The disadvantage of this method is the need for repeated washing of the precipitate, consisting of (NH_4_)_2_TiOF_5_ or (NH_4_)_3_TiOF_5_, to shift the equilibrium to the formation of Ti(OH)_4_.

In studies by [30,31], the processing of ilmenite concentrate includes fluorination of raw materials by sintering with a fluoride reagent, heat treatment of the fluorinated mass to separate fluorination products by sublimation, and pyrohydrolysis of the residue after sublimation to obtain iron oxide. In fluorination, ammonium fluoride, ammonium bifluoride, or a mixture thereof in an inert gas flow is used as a fluoride reagent, and the sublimation products are trapped with water to obtain an ammonium fluorotitanate solution. Titanium is also precipitated with aqueous ammonia. A coarse-grained precipitate of (NH_4_)_2_TiO(OH)_3_F is obtained, but TiO_2_ is in an amorphous form after calcination up to 900 °C, and anatase (95 wt%) is obtained only after maintaining it at 900 °C for 5 h. Precipitation at a concentration of (NH_4_)_2_TiF_6_, 300 g/dm^3^ with ammonia water (25% NH_3_) to pH = 9 results in the production of (NH_4_)_2_TiO(OH)_4_; after filtering and calcining at 500 °C for 2 h, the content fluorine ion in TiO_2_ is 0.3 wt%. This TiO_2_, which is anatase stable at temperatures up to 950–1000 °C, is used as a catalyst in organic synthesis processes. To obtain the rutile form of TiO_2_, it is necessary to subject the precipitate after the first filtration to repulpation and washing in ammonia water to pH = 11–12, which makes it possible to reduce the content of fluorine ion to 0.007 wt%. The process of rutilization of titanium dioxide is 99 wt% at 700 °C for 4 h, or at 800 °C for 2 h.

The proposed method for producing pigment titanium dioxide is multi-stage, energy intensive, and requires a significant consumption of ammonia.

There is a known method [32], according to which, metallic aluminum is added to a solution of fluorotitanic acid with a concentration of 250–500 g/dm^3^ TiO_2_ with stirring of the solution at the rate of 0.15–1.5% of aluminum relative to TiO_2_ in solution. In this case, aluminum acts as a pigment-rutilizing additive. Then, high-temperature pyrolysis (burning) of the solution is performed at 500–700 °C in the tower nozzle pyrolysis furnace, running on natural gas. Gaseous products containing HF are captured and disposed, with production of concentrated hydrofluoric acid. The dry product of pyrolysis—titanium dioxide containing fluorine, is pulped in water at a solid to liquid ratio of 1:3 and stirred for 1.5–2.0 h at 40–50 °C to accelerate the removal of fluorine from titanium dioxide. Milk of lime is added until 6.5–7.07 pH is reached. As a result, fluorine is bound in the form of a water-insoluble CaF_2_ that does not adversely affect paint coatings. The disadvantage of this method is the high total content of fluorine in the product.

The method of pyrolysis in two furnaces installed in series has been described in [33]. The process can be divided into two stages.

Stage I: the precipitate containing titanium salts is subject to the first hot hydrolysis at 340–400 °C for 1–3 h after removal of water by drying; typically, this stage is performed in a furnace under superheated steam and with continuous stirring. All bonds of fluorine and ammonia are broken under these conditions, with production of an intermediate product in the form of a powder consisting of TiO_2_ (95–97 wt%) and TiOF_2_.

Stage II: The first stage results in TiO_2_ containing traces of TiOF_2_ that is a grey and blue powder and, therefore, contaminates the final TiO_2_ product that should be characterized with a high degree of whiteness, in contrast. This pollutant is removed by subsequent pyrohydrolysis at 700–900 °C. The second pyrohydrolysis is usually performed in a continuously stirred oven for 60–180 min. It is advisable to introduce air and superheated steam into the furnace to complete the reaction. The end product of the second oven consists of a powder with particles of variable sizes from about 0.1 µm to about 4 µm.

Providing for the specific fineness of the TiO_2_ powder produced in this way, it can be placed on the market without further grinding.

There is a method [34] intended to obtain titanium dioxide with pyrohydrolysis of fluorammonium salts of titanium in the gas phase in the presence of water vapor. This method differs from others due to pyrohydrolysis performed by heating the reactor up to 450–500 °C at a water vapor temperature from 700 to 1200 °C, preferably 900–1000 °C, with the use of ammonium hexafluorotitanate; water vapors are obtained by burning hydrogen in oxygen in the burner, and an additional amount of water vapor, obtained by evaporation at boiling temperature, is added to their amount. However, mixtures of hydrogen and oxygen are explosive, so the method requires very strict adherence to technological regulations and safety precautions.

As the review of scientific, technical, and patent literature has shown, the use of the ammonium fluoride processing method makes it possible to successfully regenerate the used fluoride reagents. This indicates significant advantages over the sulphate method, where a large amount of dilute hydrolytic sulfuric acid is formed, contaminated with various impurities, which makes it difficult to return it back to the process. In addition, there is a danger of working with concentrated sulfuric acid during the decomposition of titanium-containing raw materials, which is accompanied by gas and reaction mass emissions. In the chlorine method, during the processing of ilmenite, at the stage of separation of titanium, silicon, aluminum, and iron chlorides, difficulties arise due to the proximity of their physicochemical properties. Additionally, from an environmental point of view, the use of chlorine in technology and the danger of phosgene formation during chlorination in the presence of carbon-containing reducing agents require strict adherence to technological regulations and safety measures. Use of the ammonium fluorine method will simplify the method for obtaining titanium dioxide from dusts of ore-thermal smelting by reducing the number of technological operations and the number of reagents with the possibility of their regeneration, improve the quality of the resulting titanium dioxide, and create the possibility of using a safer and more environmentally friendly method.

Production wastes have a complex polycomponent composition; their processing is a difficult task. The ammonium fluoride method makes it possible to separate the target components with high selectivity and obtain end products from them. Therefore, it is of interest to conduct research on ammonium fluoride processing of waste dust from the electric smelting of ilmenite concentrate, for which the process of obtaining titanium dioxide and its purification is an important stage. This work is aimed at studying titanium fluoride sublimes, the processes of their pyrohydrolysis, and purification of the resulting titanium dioxide.

## 2. Materials and Methods

Fine dust of electric smelting of ilmenite concentrate was provided by “Ust-Kamenogorsk Titanium and Magnesium Plant” JSC, with the following composition wt%: 20.57 Ti, 14.15 Fe, 12.85 Si, 0.47 Cr, and 3.42 Mn.

Analysis methods: X-ray diffraction analysis (XRD) was performed on diffractometer D8 ADVANCE “BRUKER AXS GmbH”, (Germany, Karsruhe) radiation Cu-Kα, database PDF-2 International Center for Diffraction Data ICDD (Swarthmore, PA, USA).

X-ray fluorescence analysis was performed using an Axios PANalytical spectrometer with wave dispersion (The Netherlands, Almelo).

The chemical analysis of the samples was performed using an Optima 8300 DV inductively coupled plasma optical emission spectrometer (Waltham, MA, USA, Perkin Elmer Inc.).

Experimental procedure: the initial components were thoroughly mixed in the required ratio to perform the sublimation processes for silicon fluoride and titanium. The charge sample was placed in an alundum boat and loaded into an electric furnace. Fluorination was performed in a LOP LT-50/500–1200 tubular furnace. The argon feed rate was 1.0–1.5 dm^3^/h.

A sample of titanium fluorides was placed in an alundum boat and loaded into an electric furnace during the pyrohydrolysis process. The steam feed rate was 1.5 to 2.0 dm^3^/h.

**Figure 1:**
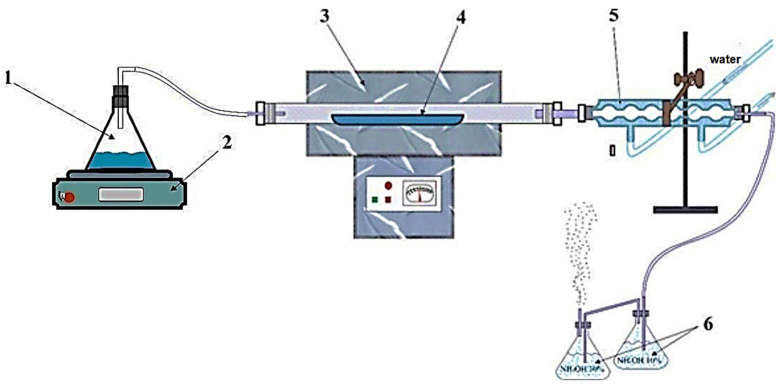
Laboratory setup for pyrohydrolysis. 1: flask with water; 2: electric stove; 3: oven LOIP LF/500–1200; 4: alundum boat; 5: refrigerator; 6: flasks with 10 wt% ammonia solution.

Experiments on pyrohydrolysis of the titanium fluorides were performed in the laboratory set shown in Figure 1.

Titanium dioxide was purified with a hydrochloric acid solution in a thermostated reactor with a volume of 0.5 dm^3^. The pulp was stirred with a glass stirrer. Stirrer rotation speed was 450 rpm.

The kinetic parameters of titanium fluoride pyrohydrolysis processes (activation energies (E_a_) and reaction rate constants (K_e_)) were calculated based on the chemical analysis data for elements in the reaction products. The degree of reaction product formation required for further calculations was determined with the following formula:

(1) $$\alpha =m/m_{calc,}$$

where *m* is the mass of the resulting product, and *m_calc_* is its theoretically possible amount. Two methods were used for calculations: the generalized Erofeev topochemical equation and the Emanuel–Knorre method.

## 3. Results and Discussion

### 3.1. Sublimates of Titanium Fluorides

Silicon fluorides were separated in the form of sublimates at the first stage of processing of fine dust obtained from the electric smelting of ilmenite concentrates; a sample of dust was mixed with ammonium bifluoride in a 1:1 ratio. The process was performed at 260 °C for 6 h [35,36,37].

Titanium fluorides were produced by processing the residue after silicon sublimation from dust at 610 °C for 2 h. The composition of titanium fluorides is shown in Table 1.

**Figure 2:**
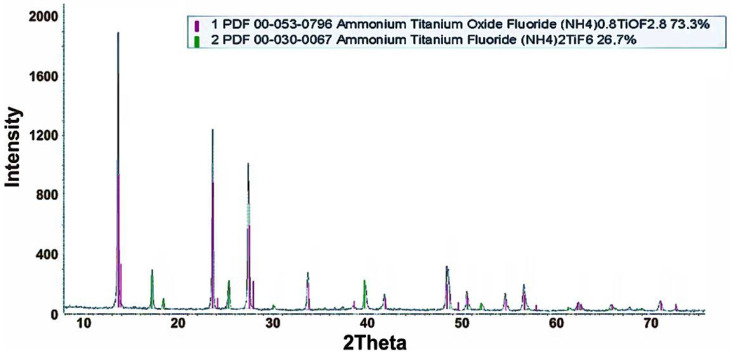
XRD pattern of the original sample of titanium fluorides.

The studied sample consists of two phases, according to XRD analysis (Figure 2). The main share is (NH_4_)_0.8_TiOF_2.8_—73.3 rel. % and (NH_4_)_2_TiF_6_—26.7 rel. %.

In [38], the process of fluorination of natural ilmenite with ammonium bifluoride was studied. According to a thermal study of the process, the compound (NH_4_)_3_Ti(OH)_0.4_F_6.6_ was initially formed, which, with increasing temperature, further decomposed stepwise, with the elimination of NH_3_ and HF molecules:

(2) $${\left({\mathrm{NH}}_{4}\right)}_{3}\mathrm{Ti}{\left(\mathrm{OH}\right)}_{0.4}F_{6.6}\to {\left({\mathrm{NH}}_{4}\right)}_{2}\mathrm{Ti}{\left(\mathrm{OH}\right)}_{0.4}F_{5.6}+{\mathrm{NH}}_{3}+\mathrm{HF}$$

(3) $${\left({\mathrm{NH}}_{4}\right)}_{2}\mathrm{Ti}{\left(\mathrm{OH}\right)}_{0.4}F_{5.6}\to {\mathrm{NH}}_{4}{\mathrm{TiO}}_{0.4}F_{4.2}↑+{\mathrm{NH}}_{3}+1.4\mathrm{HF}$$

(4) $${\mathrm{NH}}_{4}{\mathrm{TiO}}_{0.4}F_{4.2}\to {\mathrm{NH}}_{4}{\mathrm{TiO}}_{0.4}F_{4.2}↑$$

According to [38], volatile compounds of titanium TiF_4_ and ammonium oxofluorotitanate (reaction 4) with the general formula ${\mathrm{NH}}_{4}{\mathrm{TiO}}_{x}F_{5-2x}$, were formed, which incongruently sublimated, as the authors assumed, with the formation of an adduct of titanium with ammonia.

In the sublimates obtained in this work (Figure 2), according to XRD, the main component is ammonium oxyfluorotitanate, with the composition (NH_4_)_0,8_TiOF_2,8_, and (NH_4_)_2_TiF_6_ comprises a little more than a quarter. The composition of sublimates somewhat differs from the results of thermal analysis in [38]. The dusts of the electric smelting of the ilmenite concentrate differ from those of the ilmenite concentrate itself; moreover, the residues of fluorination and sublimation of silicon fluorides are used to sublimate titanium fluoride compounds. Dust contains titanium-containing phases: iron titanium oxides (Fe_2_TiO_5_ and Fe_1.5_Ti_0.5_O_3_), magnesium titanium oxide (MgTi_2_O_5_), and titanium oxide (TiO_2_) [35]. The desiliconized residues are mostly represented by such titanium-containing phases as (NH_4_)_2_TiF_6_, Ti_6_O_11_; there is also a (NH_4_)_0.8_TiOF_2.8_ phase [37]. Taking into account the results of XRD analysis of dusts and desiliconized residues of their fluorination, the process of titanium fluoride formation can be represented for the main phases of iron titanium oxides (Fe_2_TiO_5_ and Fe_1.5_Ti_0.5_O_3_), and magnesium titanium oxide (MgTi_2_O_5_), in dusts, by the following reactions:

(5) $${\mathrm{Fe}}_{2}{\mathrm{TiO}}_{5}+9{\mathrm{NH}}_{4}{\mathrm{HF}}_{2}\to {\left({\mathrm{NH}}_{4}\right)}_{2}{\mathrm{TiF}}_{6}+2{\left({\mathrm{NH}}_{4}\right)}_{3}{\mathrm{FeF}}_{6}+{\mathrm{NH}}_{3}+5H_{2}O$$

(6) $${\mathrm{Fe}}_{1.5}{\mathrm{Ti}}_{0.5}O_{3}+5{\mathrm{NH}}_{4}{\mathrm{HF}}_{2}\to 0.5{\left({\mathrm{NH}}_{4}\right)}_{2}{\mathrm{TiF}}_{6}+{\left({\mathrm{NH}}_{4}\right)}_{3}{\mathrm{FeF}}_{6}+0.5{\mathrm{FeF}}_{2}+{\mathrm{NH}}_{3}+3H_{2}O$$

(7) $${\mathrm{MgTi}}_{2}O_{5}+7{\mathrm{NH}}_{4}{\mathrm{HF}}_{2}\to 2{\left({\mathrm{NH}}_{4}\right)}_{2}{\mathrm{TiF}}_{6}+{\mathrm{MgF}}_{2}+3{\mathrm{NH}}_{3}+5H_{2}O$$

(8) $${\mathrm{Fe}}_{2}{\mathrm{TiO}}_{5}+7.4{\mathrm{NH}}_{4}{\mathrm{HF}}_{2}\to {\left({\mathrm{NH}}_{4}\right)}_{0.8}{\mathrm{TiOF}}_{2.8}+2{\left({\mathrm{NH}}_{4}\right)}_{3}{\mathrm{FeF}}_{6}+0.6{\mathrm{NH}}_{3}+4H_{2}O$$

(9) $${\mathrm{Fe}}_{1.5}{\mathrm{Ti}}_{0.5}O_{3}+4.2{\mathrm{NH}}_{4}{\mathrm{HF}}_{2}\to 0.5{\left({\mathrm{NH}}_{4}\right)}_{0.8}{\mathrm{TiOF}}_{2.8}+{\left({\mathrm{NH}}_{4}\right)}_{3}{\mathrm{FeF}}_{6}+0.5{\mathrm{FeF}}_{2}+\;+0.8{\mathrm{NH}}_{3}+2.5H_{2}O$$

(10) $${\mathrm{MgTi}}_{2}O_{5}+3.8{\;\mathrm{NH}}_{4}{\mathrm{HF}}_{2}\to \;2{\left({\mathrm{NH}}_{4}\right)}_{0.8}{\mathrm{TiOF}}_{2.8}+{\mathrm{MgF}}_{2}+2.2{\;\mathrm{NH}}_{3}+3H_{2}O$$

Trivalent iron predominates in the dust, and ferrous iron is also present in the Fe_1.5_Ti_0.5_O_3_ phase. According to the data of [38], trivalent iron present in natural ilmenite was completely reduced to divalent iron, with the release of gaseous nitrogen. However, in accordance with the results of the studies [39], the samples contained an iron-containing phase (NH_4_)FeF_6_ with ferric iron; at the same time, the studies were carried out in an argon atmosphere. Based on the foregoing, in reactions (6) and (8) iron is present in the form of two phases: (NH_4_)FeF_6_ and FeF_2_.

### 3.2. Determination of the Optimal Conditions for the Pyrohydrolysis of Titanium Fluorides

In order to obtain titanium dioxide, the sublimates obtained after fluorination and sublimation of the desiliconized residue from the ammonium fluoride treatment of dusts were subjected to pyrohydrolysis.

The pyrohydrolysis of the titanium fluorides is described by the reactions [40,41]:

(11) $${\mathrm{NH}}_{4}{\mathrm{TiOF}}_{3}+H_{2}O={\mathrm{TiO}}_{2}+{\mathrm{NH}}_{3}+3\mathrm{HF}$$

(12) $${\left({\mathrm{NH}}_{4}\right)}_{2}{\mathrm{TiF}}_{6}+2H_{2}O={\mathrm{TiO}}_{2}+2{\mathrm{NH}}_{3}+6\mathrm{HF}$$

According to [38], the pyrohydrolysis of ammonium fluorotitanates with the formation of titanium dioxide can proceed sequentially, forming intermediate compounds, according to the scheme:

(13) $${\left({\mathrm{NH}}_{4}\right)}_{2}\mathrm{Ti}{\left(\mathrm{OH}\right)}_{x}F_{6-x}\to {\mathrm{NH}}_{4}{\mathrm{TiOF}}_{3}\to {\left({\mathrm{NH}}_{4}\right)}_{0.8}{\mathrm{TiOF}}_{2.8}\;\to {\left({\mathrm{NH}}_{4}\right)}_{0.3}{\mathrm{TiOF}}_{2.3}\to {\mathrm{TiO}}_{2}$$

**Figure 3:**
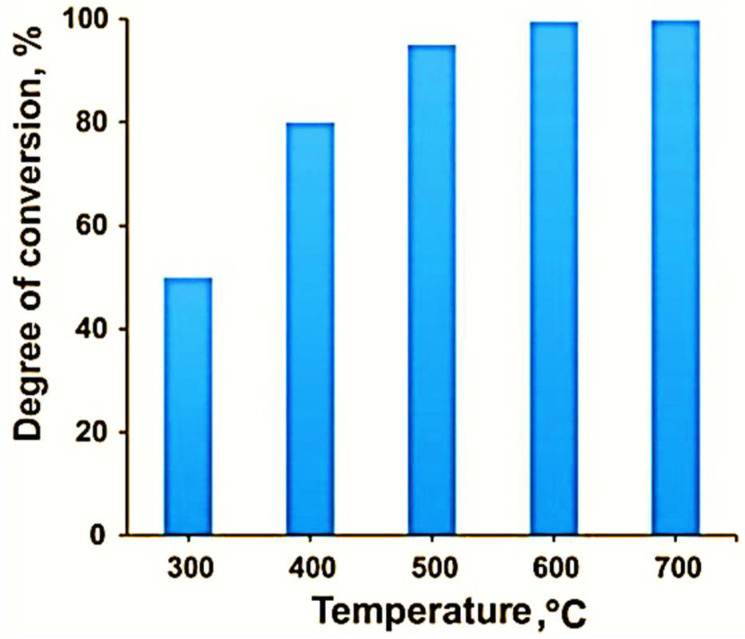
Dependence of the conversion degree of titanium fluorides into titanium dioxide on the pyrohydrolysis temperature.

**Figure 4:**
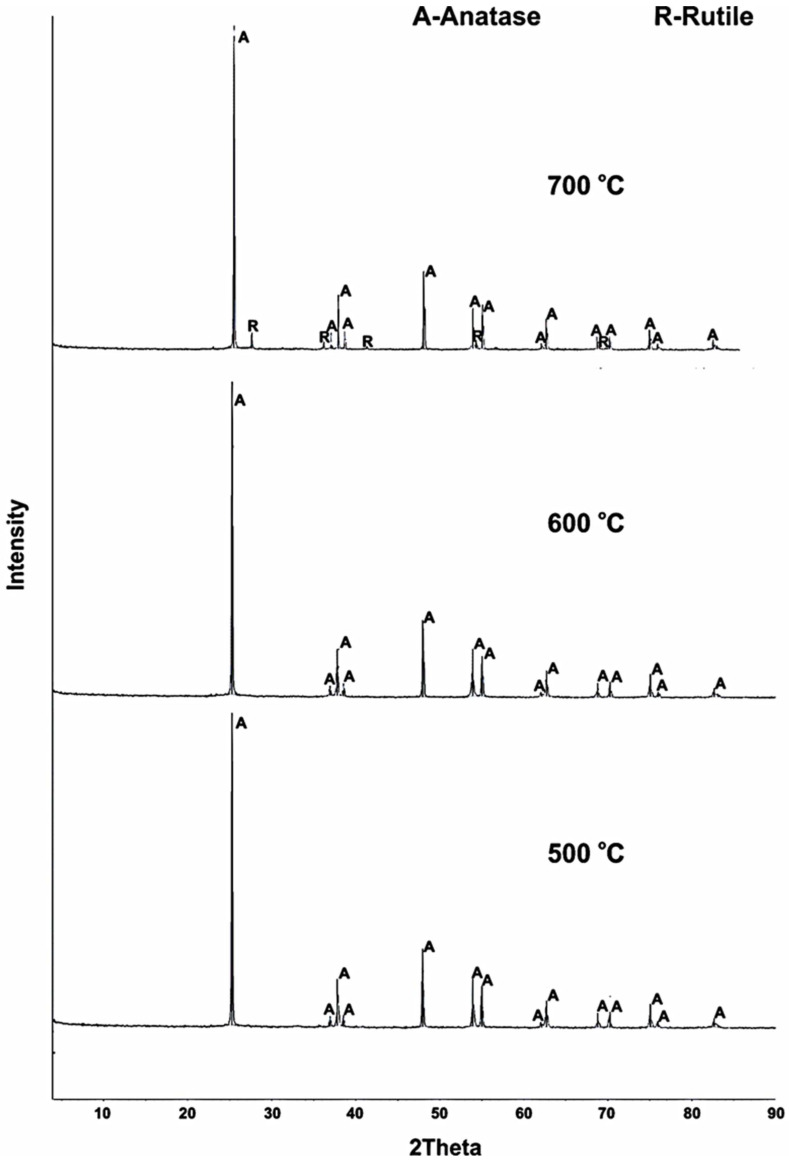
XRD patterns of titanium fluoride pyrolysis products (duration 300 min).

The effect of various parameters on the process of pyrohydrolysis has been studied. At the first stage, *the influence of the temperature of the pyrohydrolysis process* was studied. The study of the temperature effect on the degree of pyrohydrolysis of the titanium fluorides was performed in the range of 300–700 °C; the duration of the experiments was 300 min. The research results are presented in Figure 3 and Figure 4.

The data shown in Figure 3 indicate that the conversion degree of titanium fluorides into oxide during the process at 600 °C reached 99.5%, and a further increase in temperature to 700 °C did not have a significant effect since the pyrohydrolysis process was almost completely finished at 600 °C.

However, the structural modification of the resulting titanium dioxide was of interest. A series of experiments was conducted in the range of 400–700 °C in order to study the effect of the titanium fluoride pyrolysis temperature on titanium dioxide modification. The XRD analysis results show (Figure 4) that the pyrolysis of the initial product at 500–600 °C for 300 min resulted in the formation of an anatase monophase.

An increase of up to 700 °C in the process temperature resulted in the onset of the anatase → rutile transition. The proportions of anatase and rutile were 94.8 and 5.2 rel. %, respectively.

The use of pyrohydrolysis of fluoroammonium compounds for the production of titanium dioxide predetermines the presence of fluorine ions in the product, which, during the condensation of titanium oxide compounds in the temperature range of 400–700 °C and higher, apparently contributes to the formation of an anatase structure [31,42]. Depending on the final consumer destination, it is possible to subject the product of pyrohydrolysis to further processing and obtain titanium dioxide of the required modification with the other corresponding characteristics.

Thus, studies have shown that titanium fluorides are almost completely converted into titanium dioxide at 600 °C.

At the next stage, the influence of the duration of the titanium fluoride pyrohydrolysis process was studied.

**Figure 5:**
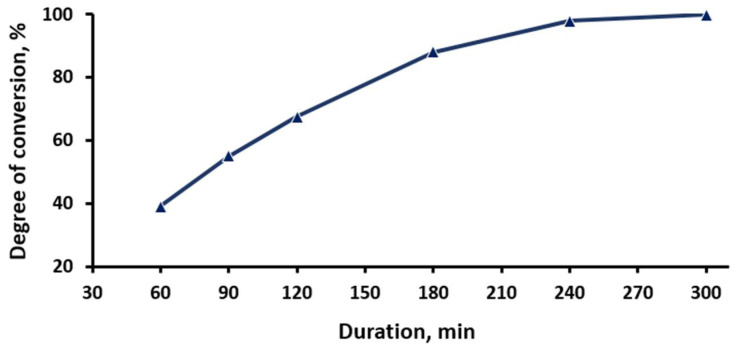
Dependence of the conversion degree of titanium fluorides into titanium dioxide on the process duration.

A series of experiments was conducted with duration of 60–300 min at 600 °C, with a steam supply rate of 1.5 dm^3^/g. The results of the experiments are shown in Figure 5.

The interval 60–240 min in Figure 5 reflects a linear dependence of the degree of conversion of titanium fluorides into oxides on the duration of pyrohydrolysis. When the process was held for 240–300 min, the pyrohydrolysis was almost totally completed, and the degree of conversion of titanium fluorides into oxides reached their maximum values. The fluoride conversion rate decreased after 240 min of the process. The composition of the pyrohydrolysis product obtained at 240 min of the process is presented in Table 2; the fluorine content in the resulting product was 0.447 wt%.

Table 2 shows that, in terms of the mass content of the main component, the resulting titanium dioxide could meet the requirements for obtaining pigment grades [43]; however, the content of impurities in the product was high and required additional processing.

The rate constants and activation energies were calculated for the pyrohydrolysis process, in accordance with reactions (11) and (12), which are presented in Table 3.

The pyrohydrolysis of titanium fluorides proceeded in the outer diffusion region, i.e., the rate of the process was limited by the insufficiently acceptable access of water vapor to titanium fluoride compounds, which must be taken into account in the further carrying out of the process to ensure sufficient mixing of the reactants.

Thus, the optimal conditions for the pyrohydrolysis of titanium fluorides are a process temperature of 600 °C and a duration of 240–300 min.

### 3.3. Purification of Titanium Dioxide from Impurities and Obtaining Rutile

Pigmented titanium dioxide is used mainly in the rutile form. Titanium dioxide produced as a result of pyrohydrolysis of titanium fluorides had a grayish tint and needed to be purified from such impurities as iron, chromium, and manganese. The oxides of these metals dissolve well in hydrochloric acid solutions; therefore, studies were carried out to purify titanium dioxide obtained as a result of pyrohydrolysis from accompanying impurities. To ensure the most complete removal of impurities from the composition of the product, the influence of the conditions for carrying out hydrochloric acid treatment was studied.

**Figure 6:**
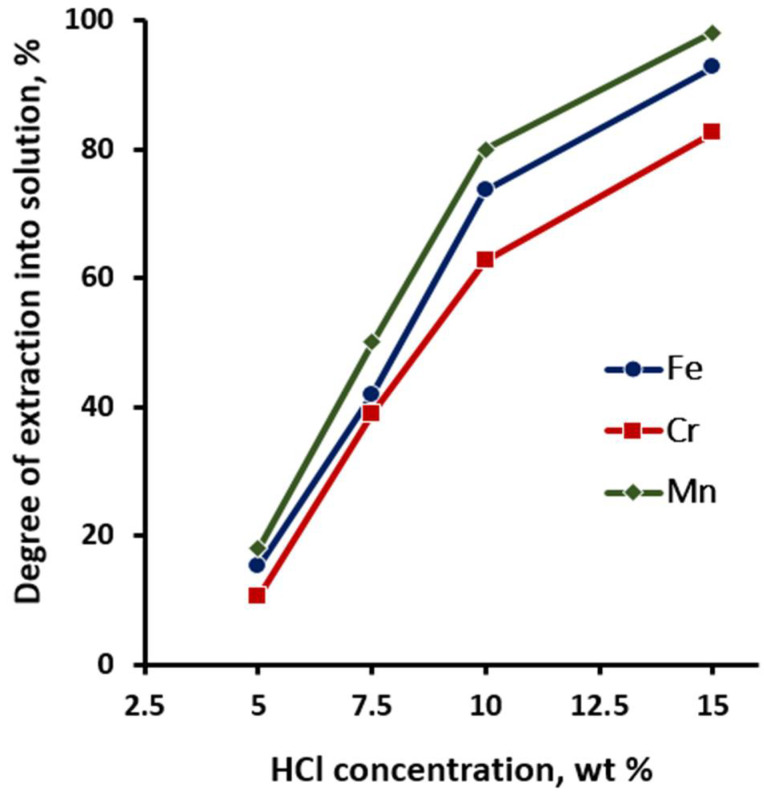
Dependence of the purification degree of titanium dioxide from iron, chromium, and manganese on the concentration of hydrochloric acid in solution.

At the first stage, *the effect of hydrochloric acid concentration* was studied. Studies were carried out with hydrochloric acid solutions with concentrations of 5, 7.5, 10, and 15 wt% while maintaining the following constant conditions: temperature 30 °C, ratio S:L = 1:8, and duration 30 min. The results of studies of this series of experiments are presented in Figure 6.

Figure 6 shows that an increase in the concentration of hydrochloric acid in the solution from 5 to 10 wt% resulted in a sharp increase in the degree of leaching of controlled impurity components into the solution of manganese from 18.0% to 80.1%, iron from 15.2% to 73.6%, chromium from 10.5% to 62.7%. A further increase in the concentration of hydrochloric acid to 15% made it possible to completely purify titanium dioxide from manganese and significantly reduce the content of iron and chromium. The optimal concentration of hydrochloric acid for the purification of titanium dioxide from impurities should be considered the range of 12.5–15 wt%.

**Figure 7:**
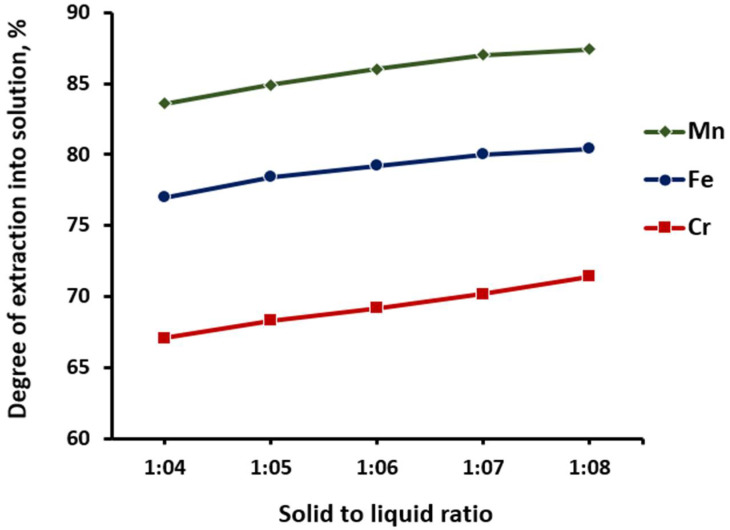
Dependence of the degree of extraction of manganese, iron, and chromium into the solution on the solid to liquid ratio.

At the next stage, *the influence of the S:L ratio on the process of impurities leaching* was studied. The effect of the ratio of the pyrohydrolysis product to the solution of 12.5 wt% hydrochloric acid at 30 °C, duration 30 min was studied. Figure 7 shows that with an increase in the ratio of S:L, there was a slight increase in the recovery of the studied impurity components into the solution, and at ratios S:L = 1:6÷8, the degree of purification of iron, manganese, and chromium reached acceptable values and amounted to ~80, ~87, and ~70%, respectively. The optimal ratios of S:L should be considered to be from 1:6 to 1:8.

**Figure 8:**
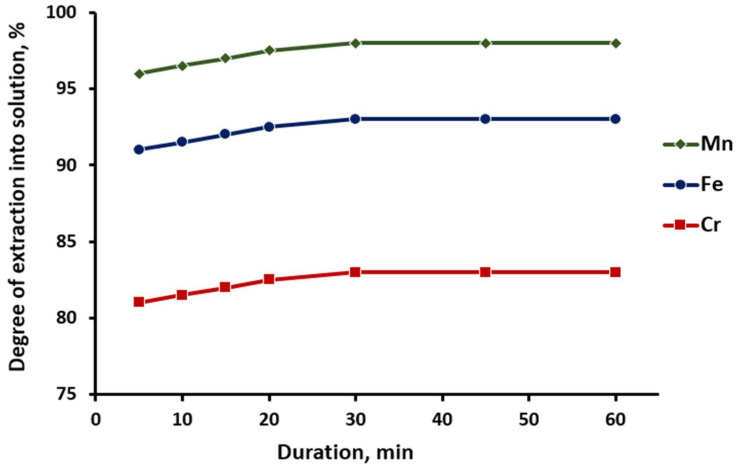
Effect of the duration of the leaching process of manganese, iron, and chromium on hydrochloric acid solution.

The next stage in studying of the effect of the duration of leaching of manganese, iron, and chromium from the pyrohydrolysis product of titanium fluorides was performed within 5–60 min at 30 °C, S:L ratio = 1:8. The results are shown in Figure 8.

Figure 8 shows that the leaching of manganese, iron, and chromium had already reached 96.1, 91.0, and 81.2%, respectively, at the initial stages of the process, and the degree of leaching reached 98.0, 93.1, and 83.1% after 30 min. The increase in the acid leaching duration did not have a significant effect. The optimal duration of the titanium dioxide purification process was determined to be 20–30 min.

**Figure 9:**
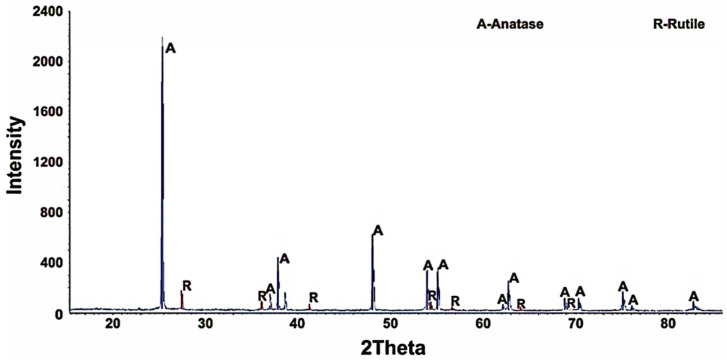
XRD pattern of titanium dioxide after treatment with hydrochloric acid.

Titanium dioxide, purified after hydrochloric acid treatment under selected optimal conditions, was studied using physical and chemical methods of analysis. According to XRD results, titanium dioxide consisted of two modifications—anatase 94.0 and rutile 6.0% (Figure 9).

**Figure 10:**
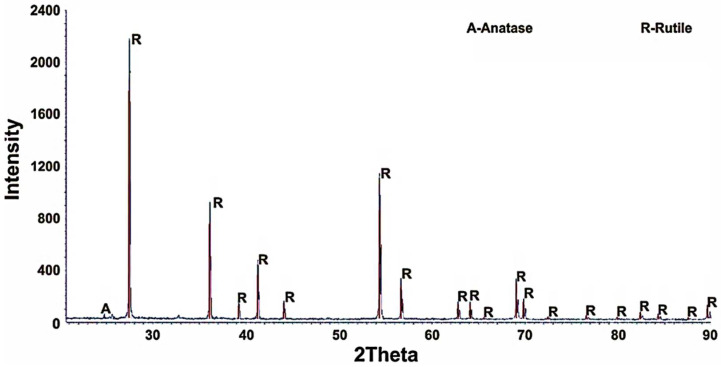
XRD pattern of rutile (900 °C, 120 min calcination time).

Titanium dioxide with an anatase structure has a wide range of applications. However, in order to obtain a rutile modification, purified titanium dioxide must be subjected to an appropriate treatment. Titanium dioxide that consists of two modifications has low pigment qualities, according to [2]. High quality is provided by rutile. In this regard, a sample of titanium dioxide with anatase base was calcined at a temperature of 900 °C during 120 min. The calcined product was studied using XRD analysis, the results of which showed that titanium dioxide was obtained with rutile modification (Figure 10).

The composition of titanium dioxide produced under optimal conditions and calcined at 900 °C is shown in Table 4.

Thus, the optimal conditions for purification of the pyrohydrolysis product of titanium fluoride and production of titanium dioxide pigment include a concentration of hydrochloric acid solution of 12.5–15 wt%, a temperature range of 25–30 °C, a solid to liquid ratio = 1:6÷8, and a duration of 20–30 min. Calcination should be performed at 900 °C for 120 min.

## 4. Conclusions

Fine dusts of ore-thermal smelting of ilmenite concentrate, containing up to 50 wt% TiO_2_ in their composition, cannot be returned back to the electric smelting process due to their high silica content (up to 20 wt%) and are a waste of titanium production.

The ammonium fluoride processing method allows for the selective separation of a valuable component from others present in the raw material.

The possibility to produce titanium dioxide from electrosmelting dusts of ilmenite concentrate was studied; while silicon was previously removed from the dusts by the ammonium fluoride method, sublimates of titanium fluorides (NH_4_)_0,8_TiOF_2,8_ and (NH_4_)_2_TiF_6_ were obtained. Titanium dioxide with the anatase structure was obtained by pyrohydrolysis of sublimates.

After purification of the product of pyrohydrolysis of titanium fluorides from impurities of iron, chromium, and manganese using hydrochloric acid solutions, pigmentary titanium dioxide of rutile modification with a content of 99.8 wt% TiO_2_ was obtained.

## Figures and Tables

**Table 1:** Content of controlled components in titanium fluorides, wt%.

Ti	Fe	Si	Cr	Al	F	O
58.99	0.56	0.005	0.095	0.140	20.377	19.76

**Table 2:** The content of the main components in the pyrohydrolysis product of titanium fluorides (600 °C, 5 g), wt%.

TiO_2_	Fe_2_O_3_	Cr_2_O_3_	Al_2_O_3_	SiO_2_	F
98.5	0.807	0.183	0.266	0.012	0.447

**Table 3:** The values of the rate constants and activation energies of the processes described by reactions (11) and (12).

Reaction (11)		Reaction (12)	
Ke, s^−1^	E_a_, kJ/mol	Ke, s^−1^	E_a_, kJ/mol
16.1 × 10^−5^	14.7	12.9 × 10^−5^	15.3

**Table 4:** The content of the main components in pigmentary titanium dioxide, wt%.

TiO_2_	SiO_2_	Cr_2_O_3_	MnO_2_	Fe_2_O_3_	F
99.8	0.0005	0.032	0.005	0.039	n/d

## Data Availability

The data and results presented in this study are available in the article.

## Abbreviations

IMOB JSC	Institute of Metallurgy and Ore Beneficiation Joint Stock Company
UKTMP JSC	Ust-Kamenogorsk Titanium and Magnesium Plant Joint-Stock Company
XRD	X-ray phase analysis
Solid to liquid ratio (S:L)	the ratio of the weight of the solid phase (in grams) to the volume of the liquid phase (in mL)
